# Detection of sexually transmitted infections among transvestites and transsexual women in prison in the metropolitan region of Rio de Janeiro, Brazil

**DOI:** 10.1590/1980-549720230058

**Published:** 2023-12-11

**Authors:** Carlos Renato Alves-da-Silva, Claudia Bonan, Saint Clair dos Santos Gomes, Rosilene Santarone Vieira

**Affiliations:** I Fundação Oswaldo Cruz. Instituto Nacional de Saúde da Mulher, da Criança e do Adolescente Fernandes Figueira – Rio de Janeiro (RJ), Brazil.; II State Secretariat for Penitentiary Administration, LGBTI Health and Citizenship Support Division – Rio de Janeiro (RJ), Brazil.

**Keywords:** Transgender persons, Prisons, Syphilis, Hepatitis, HIV seropositivity, Pessoas transgênero, Prisões, Sífilis, Hepatite, Soropositividade para HIV

## Abstract

**Objective:**

To evaluate the seropositivity rate of rapid tests for HIV, syphilis and hepatitis B and C among transvestites and transgender women (transfeminine persons) inmates in the metropolitan region of Rio de Janeiro, analyzing the results based on sociodemographic, prison profile and access to health technologies to prevent sexually transmitted infections (STIs).

**Methods:**

Cross-sectional census-type study carried out with transfeminine in eleven male prisons in Rio de Janeiro, between the months of April and June 2021.

**Results:**

The detection rates found were 34.4% for HIV, and 48.9% for syphilis, and 0.8% for type B and C hepatitis. Seropositivity for more than one infection was verified in 25.4% of participants, and HIV/syphilis was the most prevalent. An increase in the level of education (p=0.037) and having a steady partner in prison (p=0.041) were considered protective factors for STIs in this population. Difficulties were identified in accessing STI prevention technologies, such as male condoms, lubricating gel, rapid tests, and prophylactic antiretroviral therapies for HIV.

**Conclusion:**

HIV and syphilis seropositivity rates were high, but within the profile found in this population in other studies inside or outside prisons. The data found indicates the need to incorporate effective strategies for access to health technologies for the prevention of STIs. The scarcity of scientific publications containing epidemiological data on STIs in the transfeminine prison population limited deeper comparisons of the results obtained in this study.

## RESUMO

**Objetivo:**

Avaliar a taxa de soropositividade dos testes rápidos para HIV, sífilis e hepatite B e C entre travestis e mulheres transexuais (transfemininas) privadas de liberdade na Região Metropolitana do Rio de Janeiro, analisando os resultados diante do perfil sociodemográfico, prisional e acesso às tecnologias de saúde para prevenir infecções sexualmente transmissíveis (IST).

**Métodos:**

Estudo transversal do tipo censitário realizado com transfemininas em 11 prisões do Rio de Janeiro, entre os meses de abril e junho de 2021.

**Resultados:**

As taxas de soropositividade encontradas foram de 34,4% para o HIV, 48,9% para sífilis e 0,8% para as hepatites do tipo B e C. A soropositividade para mais de uma infecção foi verificada em 25,4% das participantes, e HIV/sífilis foi a mais prevalente. O aumento no nível de escolarização (p=0,037), e possuir parceiro fixo na prisão (p=0,041) foram considerados fatores de proteção para as IST nessa população. Foram identificadas dificuldades no acesso às tecnologias de prevenção contra IST, como preservativo masculino, gel lubrificante, testes rápidos e terapias antirretrovirais profiláticas para o HIV.

**Conclusão:**

As taxas de soropositividade para o HIV e sífilis foram elevadas, mas no perfil encontrado nessa população em outros estudos dentro e fora das prisões. Os dados encontrados indicam a necessidade de incorporar estratégias efetivas para o acesso às tecnologias em saúde para a prevenção das IST. A escassez de publicações científicas contendo dados epidemiológicos sobre IST na população transfeminina em situação de prisão limitou a realização de comparações mais profundas dos resultados obtidos neste estudo.

## INTRODUCTION

The majority of prison spaces in Brazil are reported due to prison overcrowding, inadequate physical structure, deficiencies in human resources, and specialized materials for health assistance and promotion, among other situations mentioned in publications that highlighted the potential of these spaces as human rights violators^1,2^.

Imprisonment conditions of specific groups, such as women, the aged, indigenous people, foreigners, people with disabilities, and people self-declared as lesbian, gay, bisexual, transgender, and intersex (LGBTI), are marked by the exacerbation of these violations, in the face of non-compliance of the duty of care inherent to the vulnerabilities established by each specific group, as defined in some national and international regulations^3–5^.

Social vulnerability among people from the LGBTI population is not homogeneous. It is observed that transvestites and transsexual women, also defined as transfeminine people, make up a target group for greater discrimination and violence, both due to the difficulties experienced in educational establishments and the lack of opportunities in the formal job market and due to their very existence, since they live in the country with the highest transgender death rate in the world^6^.

Some studies show the conditions of deprivation of liberty of transfeminine women, revealing scenarios of transphobic practices and violations of these people's rights to citizenship and health^7,8^, such as respect for the use of their social names, the use of clothes and accessories according to their gender, the guarantee of hormone treatment, access to and continuity of their education, among other provisions described in the National Policy for Comprehensive Health of the LGBT Population^9^ and in the legislation that provides for the treatment of these people in the Brazilian penitentiary system.

The National Penitentiary Department (*Departamento Penitenciário Nacional* – Depen) recently published a population survey, referring to the second half of 2022, recording that, in Brazil, there were 830 thousand people deprived of liberty (PDL), however, of this total, 650 thousand were serving their criminal sentences in prison units and the rest of them were under house arrest. This census also revealed that around 12,500 self-declared LGBTI people were imprisoned in Brazilian prison units^1^, of which approximately 1,600 were transvestites and transsexual women. The methodology adopted in this survey to quantify this population was based on completing a questionnaire sent by Depen to the bodies of all federative units responsible for the penitentiary system.

The prison population in the state of Rio de Janeiro (RJ), recorded in the same publication, revealed that around 48 thousand people were in RJ's prisons, among which 579 declared themselves LGBTI, with 176 transvestites and transsexual women being deprived of liberty, occupying second place in the national ranking, in absolute number, in custody of the transfeminine population^10^.

A technical document, published by the Ministry of Women, Family and Human Rights, in 2020, containing the national diagnosis of the criminal treatment of this population in Brazil, revealed that the imprisonment of transfeminine women in our country occurs, for the most part, in prisons aimed at male inmates and, in large part, at an alarming disproportion, with them being a minority of the total number of people imprisoned^11^. These conditions drastically affect the overall health of these people, whether due to possible psychological damage or harm to their physical health.

Health care access and quality in the Brazilian penitentiary system is one of the greatest challenges to be faced. The National Policy for Comprehensive Penitentiary Health Care (*Política Nacional de Atenção Integral à Saúde Penitenciária* – Pnaisp) has emerged as a powerful management strategy to resolve these obstacles in several Brazilian states. This policy aims to guarantee access to comprehensive primary health care by PDL^12,13^. The implementation of Pnaisp, in RJ, intensified, from January 2022, with the accession of the capital of Rio de Janeiro, however, during the period if this study, only four units, of the total of 46 prison units with outpatient care, had Pnaisp teams^14^.

The spread of sexually transmitted infections (STIs) in prisons is one of the problems to be tackled by prison health teams, faced with environments with conditions conducive to unsafe sexual practices, as described in national and international studies^15–18^. However, it is necessary to know and analyze this reality in order to promote effective actions to reduce the risks of STIs, such as combined strategies, in order to improve access to condoms, HIV prophylactic therapies, rapid STI testing regularly every six months, among other actions recommended by the Ministry of Health (MoH)^19^.

This study aimed to evaluate the isolated and combined seropositivity rate for HIV, syphilis, and type B and C hepatitis, through the use of rapid tests among transfeminines deprived of liberty, in the Metropolitan Region of Rio de Janeiro and to identify potential associated factors, considering sociodemographic characteristics, prison conditions and access to health technologies for the prevention of STIs, such as male condoms, lubricants, and rapid tests.

## METHODS

### Study design

This is a cross-sectional study with a quantitative approach in a census format, that is, involving the entire self-declared adult transfeminine population deprived of liberty in the Metropolitan Region of Rio de Janeiro.

### Background

**Chart 1:**
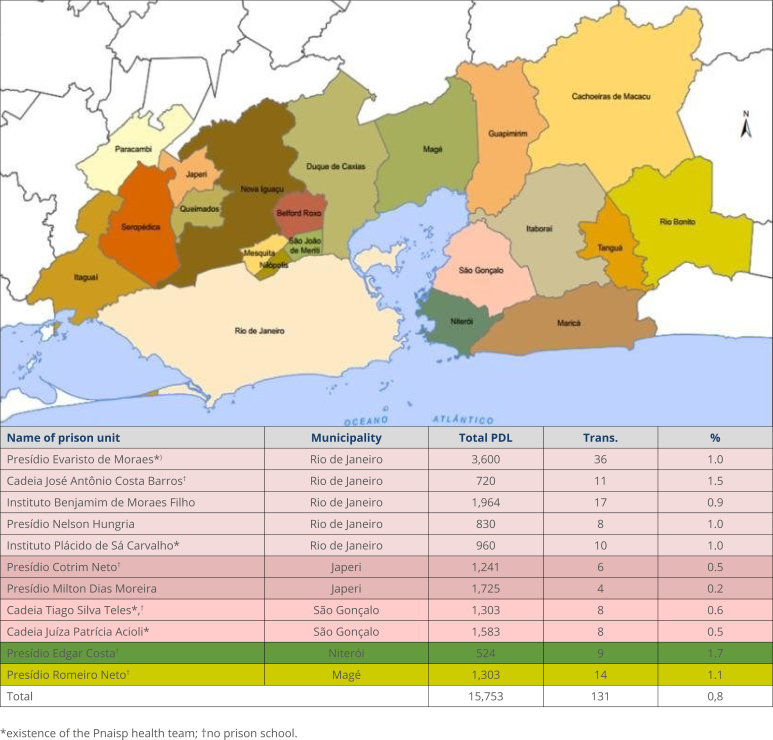
Map of the Metropolitan Region of Rio de Janeiro and the selected prison units with their respective population data, 2021.

Source: Seap-RJ, 2021.

Eleven male prisons that held transfeminine people during the study period, with their respective locations, total prison population, number of transfeminine participants, and the percentage rate between participants and the total prison population (Chart 1). It is clarified that, during the study period, there were no transfeminine prisoners in female prison units and that the selected units are classified as neutral, to which LGBTI people are sent in RJ.

### Participants

Transfeminine PDL were previously selected, by consulting the Penitentiary Identification System (*Sistema de Identificação Penitenciária* – Sipen) of the State Secretariat for Penitentiary Administration (*Secretaria de Estado de Administração Penitenciária* – Seap-RJ), a nominal database updated daily, through information about the people entering the RJ penitentiary system. Access to this database was possible, as one of the researchers is a public server pharmacist who held the position of director of the LGBTI Health and Citizenship Support Division at Seap (RJ). The study also adopted the “snowball” method, to identify possible transfeminines not included in Sipen^20^, when the selected participant reported the existence of transfeminines not registered in this database, being subsequently invited to participate in the research. All people who declared themselves transfeminine were included, using terms such as transvestite, tranny, transsexual woman, trans, transex, woman or any reference to transfeminine identity. Tran feminine women who had been in prison for less than six months were excluded, the time period recommended by the MoH for rapid testing among transvestites and transsexual women in prison^19^. Interviewees who did not have the results of rapid tests in the last six months in their medical records and who expressed opposition to carrying out rapid testing after the interview were also excluded.

### Data collect

Data collect was carried out in the period between April and June 2021, using a structured questionnaire containing questions with closed answers and selecting people who declared themselves transvestites or transsexual women. This information allowed to describe the sociodemographic and prison profiles, as well as access to health technologies used to prevent STIs: male condoms, lubricants, and rapid testing. However, the variables related to length of stay and existence of registered visitors were extracted from Sipen, a strategy adopted to improve data accuracy. Data relating to the results of serological tests for HIV, syphilis, and hepatitis B and C in the last six months, as recommended by the MoH, were extracted from the health records of the interviewees. When this information was not available, rapid tests were requested to be carried out by the prison unit's health team, and no situation of rapid test shortages, inaccessibility to health records or refusal by health professionals to carry out rapid tests were observed. These professionals are responsible for inputting the results in medical records, as well as confirming the results, through confirmatory laboratory tests, according to standardized operational procedures established for each infection. The rapid tests recorded in the medical records and carried out after the interviews were based on immunochromatography technology, using whole blood finger-prick test.

All rapid tests were made available to prison units in Rio de Janeiro, through the State Secretariat of Health of Rio de Janeiro (*Secretaria de Estado da Saúde do Rio de Janeiro* – SES-RJ) and by the municipal Health Departments of the respective cities where the prison units were located, and which had health teams hired by Pnaisp.

### Study variables

The sociodemographic variables analyzed were: age group in years (from 18 to 29, from 30 to 39, over 40); race/color (white, non-white); education (incomplete and complete elementary school, incomplete and complete high school, higher education); sexual orientation (heterosexual, bisexual); paid activity before prison (not working, sex worker, formal or other informal employment); and use of illicit substances before arrest (yes, no). The prison variables were: length of stay in prison (up to one year, over one year); proportion between transfeminines and men in the same cell (from 1/1 to 1/10, over 1/10); steady partner in prison (yes, no); number of partners in prison (up to one, more than one); registered visitor (yes, no); work activity in prison (yes, no); schooling in prison (yes, no). The health care technologies were: access to male condoms (yes, no); access to lubricant (yes, no); carrying out rapid tests in the last six months (yes, no); and rapid test positivity (yes, no). The variables analyzed were considered factors associated with STIs, with some being used in some studies^15,21^.

### Data analysis

Collected data were analyzed using the statistical program Jasp (Jeffrey's Amazing Statistics Program) version 0.17.1.0, evaluating percentage analyses of sociodemographic, prison and access to health technology variables, as well as the results for each rapid STI test evaluated. The variable called “positive result in at least one of the STI tests” was created to evaluate associations with sociodemographic variables, prison conditions, and access to health care technologies dichotomized to find statistical significance. The chi-square test was applied in all analyses, with a statistical significance level of 5% (p<0.05).

### Ethical aspects

The research was duly approved by the Research Ethics Committee of the Fernandes Figueira National Institute of Women, Children and Adolescent Health (*Instituto Nacional de Saúde da Mulher, da Criança e do Adolescente Fernandes Figueira* – IFF-Fiocruz), under CAAE No. 36338520.2.0000.5269, authorized by the Criminal Executions Court of the Court of Justice of the state of Rio de Janeiro and by the School of Penitentiary Management of Seap-RJ, through Process SEI-21/087/000987/2019. All ethical and safety issues involved in research with PDL were observed, including informed consents signed by all study participants. All interviewees with positive rapid test results were assisted by the prison unit's health teams, being included in institutional clinical protocols for the treatment of STIs.

## RESULTS

151 self-declared transfeminine people were identified, however 13 people had been in prison for less than six months and seven did not consent to rapid tests for the STIs analyzed in this study. The database identified 92 of the selected transfeminines (60.1%), the other interviewees were reached by the snowball method. Recruitment of interviewees was carried out in 11 prison units (Chart 1), and it was found that four prison units had health teams, through the implementation of Pnaisp, and five prison units had prison schools. Data relating to the sociodemographic and prison profile of the interviewees were collected from a sample composed of 131 transfeminines (Table 1), demonstrating that 85 were young adults under the age of 29 (64.9%), and, not unlike the Brazilian prison population, the sample consisted of 113 black and brown women (86.3%). Education demonstrated that only 25 interviewees managed to complete high school education (19.1%) and 65 did not complete elementary education (49.6%). The majority, that is, 123 transfeminines, revealed that they were heterosexual (93.9%). Prostitution was the most cited source of income for 55 of the interviewees (42.0%), and the use of illicit substances before imprisonment was revealed by 106 transfeminines (80.9%). The situation inside prisons also revealed that 72 interviewees had been in prison for more than a year (55.0%) and 111 were incarcerated with cisgender and heterosexual men, in a proportion greater than 1/10 (84.7%). It was revealed that 109 of the interviewees had relationships with two or more partners throughout their imprisonment (83.2%) and, at the time of the interviews, 77 revealed that they were in a steady relationship (58.8%). Among those interviewed, 66 did not have registered visitors (50.4%) and few were involved, or had been involved in resocializing practices, with records that 23 were working or have worked in prisons (17.6%) and only 26 had the opportunity to enter prison schools (19.8%).

**Table 1:** Socioeconomic, demographic, and prison characteristics of transfeminines deprived of liberty in the Metropolitan Region of Rio de Janeiro, Brazil, 2021.

Characteristic – socioeconomic		n	%
Age range (years)			
	18–29	85	64.9
	30–39	34	25.9
	40 and over	12	9.2
Race/color			
	Non-white	113	86.3
	White	18	13.7
Education			
	Incomplete elementary school	65	49.6
	Complete elementary school	4	3.0
	Incomplete High School	37	28.3
	Complete High School	19	14.5
	Complete and incomplete higher education	6	4.6
Sexual orientation			
	Heterosexual	123	93.9
	Bisexual	8	5.3
Paid activity before imprisonment			
	Was not working	23	17.6
	Sex worker	55	42.0
	Formal or informal employment (beauty, fashion, food, and others)	53	40.4
Use of illicit substances before the current arrest			
	Yes	106	80.9
	No	25	19.1
Characteristic – prison			
Length of stay in prison (years)			
	Up to 1	59	45.0
	More than 1	72	55.0
Proportion between transfeminines and men in the cell			
	1/1 to 1/10	20	15.3
	Over 1/10	111	84.7
Steady partner in prison			
	Yes	77	58.8
	No	54	41.2
Number of partners in prison			
	Up to 1	22	16.8
	More than 1	109	83.2
Registered visitor			
	Yes	65	49.6
	No	66	50.4
Labor activity in prison			
	Yes	23	17.6
	No	108	82.4
Education in prison			
	Yes	26	19.8
	No	105	80.2

N sample: 131 people.

The results on access by transfeminines to male condoms, lubricants, and rapid testing for STIs (Table 2) demonstrated that 29 had difficulty accessing male condoms (22.1%) and 95, lubricants (72.5%). It was found that 37 subjects were on antiretroviral therapy (ART) for HIV (28.2%), thus, 94 health records were consulted to evaluate the performance of rapid testing (71.8%), in which 41 interviewees were tested for HIV (43.6%), among whom, three were positive for the rapid HIV test (2.3%). Among the 53 who underwent rapid testing after the interview, five tested positive for HIV (9.4%). The total of these results revealed that 45 of the transfeminines tested positive for HIV (34.4%). At the time of the interview, none were observed undergoing treatment for syphilis, and the syphilis testing profile revealed that 56 transfeminines had records of testing in the last six months (42.8%). The results of the rapid test for syphilis in the last six months from the date of the interview revealed that 56 were positive for this infection (69.6%), while 25 of them had positive results after the interview (33.3%). Thus, among them, it was found that 64 people were positive for syphilis (48.9%). The testing profile for hepatitis types B and C proved to be identical for both infections, since, throughout this study, it was verified that two interviewees were already undergoing treatment for these infections, one for hepatitis B (0.8%) and one for hepatitis C (0.8%). No positive results were found in the 55 medical records with records of testing for hepatitis in the last six months (42.0%), as well as no positive results in the 75 rapid tests carried out after the interviews (57.2%). The serological STIs profile through rapid testing of the interviewees revealed that 71 had positive results for at least one of the four STIs tested in this study (54.2%). Among these 71 transfeminines, it was found that 19 presented more than one positive result in the rapid tests carried out (26.8%), in which the most prevalent combined seropositivity was HIV/syphilis, which accounted for 18 of the cases (94.7%). The associations made between the results of rapid tests and sociodemographic, prison characteristics and access to health care technologies used to prevent STIs (Table 3) demonstrated that, among the 46 transfeminine women over the age of 29, 31 had positive results for STIs (67.4%), compared to 85 aged between 18 and 29 years, in which there were 40 positive results (47.1%); (p=0.026). 44 positive tests for STIs were revealed among the 70 interviewees who only attended elementary school (62.8%), compared to 27 positive results among the 61 who attended high school and higher education (44.3%); (p=0.033). Of the 54 who declared not having a steady partner, 35 had more positive results for STIs (64.8%), compared to the 36 positive results among the 77 who reported having emotional stability (46.8%); (p=0.041).

**Table 2:** Access to health technologies associated with sexually transmitted infections and rapid test results among transfeminine women deprived of liberty in the Metropolitan Region of Rio de Janeiro, Brazil, 2021.

Characteristic		n	%
Access to male condoms			
	Yes	29/131	22.1
	No	102/131	77.9
Access to lubricants			
	Yes	95/131	72.5
	No	36/131	27.5
Carrying out the rapid test for HIV			
	No, subject already undergoing treatment	37/131	28.2
	Registered in the medical record, with a positive result	3/131	2.3
	Registered in the medical record, with a negative result	38/131	29.0
	Carried out during the interview, negative result	48/131	36.6
	Carried out during the interview, positive result	5/131	3.9
Carrying out the rapid test for syphilis			
	No, subject already undergoing treatment	0/131	0.0
	Registered in the medical record, with a positive result	39/131	29.8
	Registered in the medical record, with a negative result	17/131	13.0
	Carried out during the interview, negative result	50/131	38.1
	Carried out during the interview, positive result	25/131	19.1
Carrying out the rapid test for hepatitis B			
	No, subject already undergoing treatment	1/131	0.8
	Registered in the medical record, with a positive result	0/131	0.0
	Registered in the medical record, with a negative result	55/131	42.0
	Carried out during the interview, negative result	75/131	57.2
	Carried out during the interview, positive result	0/131	0.0
Carrying out the rapid test for hepatitis C			
	No, subject already undergoing treatment	1/131	0.8
	Registered in the medical record, with a positive result	0/131	0.0
	Registered in the medical record, with a negative result	55/131	42.0
	Carried out during the interview, negative result	75/131	57.2
	Carried out during the interview, positive result	0/131	0.0
Positive result in at least one of the STI tests			
	Yes	71/131	54.2
	No	60/131	45.8
Frequency of positive results for STIs			
	Only one STI	52/71	73.2
	HIV/syphilis	18/71	25.4
	HIV/hepatitis B	1/71	1.4

STI: sexually transmitted infections; HIV: human immunodeficiency virus.

**Table 3:** Association between the results of rapid testing for sexually transmitted infections and sociodemographic, prison characteristics and access to health technologies among transfeminine women deprived of liberty in the Metropolitan Region of Rio de Janeiro, Brazil, 2021.

Characteristic		Positive result for STI					
		N	Yes		No		p-value
			n	%	n	%	
Age range (years)							
	19–29	85	40	47.1	45	52.9	**0.026**
	Over 29	46	31	67.4	15	32.6	
Race/color							
	White	113	62	54.9	51	45.1	0.700
	Non-white	18	9	50.0	9	50.0	
Education							
	Elementary school	70	44	62.8	26	37.2	**0.033**
	High School and Higher education	61	27	44.3	34	55.7	
Sexual orientation							
	Heterosexual	123	66	53.7	57	46.3	0.627
	Bisexual	8	5	62.5	3	37.5	
Paid activity before imprisonment							
	Sex worker	55	30	54.5	25	45.5	0.946
	Formal or other informal employment	76	41	53.9	35	46.1	
Use of illicit substances before imprisonment							
	Yes	106	61	57.5	45	42.5	0.113
	No	25	10	40.0	15	60.0	
Length of stay in prison (years)							
	Up to 1	59	34	57.6	25	42.4	0.476
	More than 1	72	37	51.4	35	48.6	
Proportion between trans. and men in the cell							
	1/1 to 1/10	20	11	55.0	9	45.0	0.938
	Over 1/10	111	60	54.1	51	45.9	
Steady partner in prison							
	Yes	77	36	46.8	41	53.2	**0.041**
	No	54	35	64.8	19	35.2	
Number of partners in prison							
	Up to 1	22	13	59.1	9	40.9	0.614
	More than 1	109	58	53.2	51	46.8	
Registered visitor							
	Yes	65	30	46.2	35	53.8	0.067
	No	66	41	62.1	25	37.9	
Education in prison							
	Yes	26	15	57.7	11	42.3	0.690
	No	105	56	53.3	49	46.7	
Access to male condoms							
	Yes	102	55	53.9	47	46.1	0.905
	No	29	16	55.2	13	44.8	
Access to lubricants							
	Yes	36	16	44.4	20	55.6	0.168
	No	95	55	57.9	40	42.1	

N sample: 131 people. Bold indicates statistically significant p-values. STI: sexually transmitted infections.

## DISCUSSION

The observation that more than half of the medical records checked did not have records of regular rapid testing for STIs demonstrated the fragility of the health care offered to this population. The detection rate for HIV was similar when compared with other national studies with this population outside prisons^22,23^ and with a study carried out in Mexico^24^. The majority of interviewees were young adults, black or brown, with low education, and with prostitution as their main source of income before imprisonment. A higher level of education, having a steady partner in prison, and younger age proved to be protective factors for the STIs analyzed.

The absence of records of rapid testing in the medical records can be translated into the difficulties in accessing health services reported in the various studies with the transfeminine population, both due to the technical lack of preparation of the health professionals who assist these people and the Brazilian penitentiary system itself, an environment that enhances violations of the health rights of the population deprived of liberty^25,26^.

The HIV detection rate of 34.4% found in this study, was similar to that found in 2012 in Mexico City^24^, where 31.9% of incarcerated transfeminines tested positive for HIV in rapid tests. In the systematic review with meta-analysis published in 2018^27^, which searched for studies on STIs among key incarcerated populations, only two articles were found, one carried out in the United States of America and the other in Argentina, signaling the scarcity of studies with this population in the prison environment, as already recorded in research published in 2019^28^. The few epidemiological data published on HIV and the lack of data for syphilis and hepatitis B and C in the transfeminine population in prisons were limiting factors for comparative purposes. The sociodemographic and prison profile demonstrated a known situation in relation to transfeminines in prison^7,29,30^, a reality composed of factors associated with trans necropolitics, such as early family separation, difficulties in remaining in school environments and accessing the labor market, prostitution on the streets, surrounded by drugs and theft, facilitating conditions that lead them to prison or death in the broadest sense^31^. The conditions of incarceration observed in this study, in which transfeminines share spaces with cisgender and heterosexual men, are comparable to those portrayed in a study carried out in the state of São Paulo^8^ and in the national report published in 2020, situations favorable to sexual practices motivated by several factors, both due to economic favoritism and emotional relationships^11^.

The abandonment of this population in prisons occurs in similar proportions to cisgender women. As reported in the study carried out in 2019, around 60% of women incarcerated in Brazilian prisons did not receive visits from family members^32^. Family abandonment among transfeminines begins, in most cases, before prison, a situation that often leads them to prostitution, which, despite being a recognized profession in Brazil, cannot be the only fate for these people^6,33,34^.

Schooling was considered a protective factor for STIs, as demonstrated in other studies with the transfeminine population outside prisons^35^, however, the factors that indicated lower detection rates for these infections among younger transfeminines and those with a steady partner require further studies to purposes of comparisons and extrapolations. The 5.1% refusal to undergo rapid STI tests observed in this study was higher than that found in a study carried out in 2007 with cisgender women in prison in São Paulo, when 3% of them did not agree to participate in the tests^36^. It is important to highlight that, often, the refusal to carry out rapid tests for STIs, especially for HIV, is directly related to the fear of receiving difficult news and the stigma of the repercussion of this result within the prison unit, whether among other transfeminine women by pride and power^37^, or by the violence that can be perpetrated by their sexual partners. Ther fore, it is necessary for health teams to have strategies for an appropriate approach, evaluating the psychological and physical consequences of carrying out rapid STI tests inside prisons. More than revealing epidemiological data, it is necessary to understand social dynamics, knowledge, attitudes, and practices established within Brazilian prisons to adopt effective preventive measures aimed at mitigating the spread of STIs, which make transfeminine women even more vulnerable, as assessed in the systematic review published in 2019^38^. It is essential that prison health teams can adopt combined prevention actions to combat these diseases, through educational campaigns on measures to prevent and treat these infections, regular rapid testing and incorporation and promotion of access to prophylactic technologies to the risk of STIs. And, above all, that these actions can accommodate the specific health demands of this population in the prison reality.
